# Enhanced Goat Milk MUFA Quality *via* Date Pit Supplementation: A Time-Based Pattern Recognition Analysis Utilizing Agricultural Waste Byproduct

**DOI:** 10.1155/2023/1797017

**Published:** 2023-05-31

**Authors:** M. R. Abd Rahman, Z. Hassan, M. S. Hassan, R. Hashim, L. S. Wong, W. Y. Leong, S. H. Syd Jaafar, S. Salvamani

**Affiliations:** ^1^Faculty of Health and Life Sciences, INTI International University, Persiaran Perdana Bandar Baru Nilai, Putra Nilai, 71800 Nilai, N. Sembilan, Malaysia; ^2^Department of Food Biotechnology, Faculty of Science and Technology, Universiti Sains Islam Malaysia, Bandar Baru Nilai, 71800 Nilai, Negeri Sembilan, Malaysia; ^3^Department of Industrial Chemistry, Faculty of Science and Technology, Universiti Sains Islam Malaysia, Bandar Baru Nilai, 71800 Nilai, Negeri Sembilan, Malaysia; ^4^School of Health Sciences, International Medical University, Bukit Jalil, 57000 Kuala Lumpur, Malaysia

## Abstract

Date pits are agricultural waste byproducts and are available in tons yearly. Milk MUFAs are lipids beneficial for health and sorted out for food product development. This work is aimed at researching the effect of supplementing dairy goats with date pit powder (DPP) as a source of fatty acids (FA), an alternative to enhancing the unsaturated FA in milk and analysed *via* chemometrics in a 3-month supplementation-based study. Saanen-Boer crossed dairy goats were divided into six groups comprising of control, 10 g and 20 g both for Ajwa DPP (high-quality dates) and Mariami DPP (agricultural waste byproduct), and another 30 g for Mariami DPP only. The supplementation exercise was done daily on each dairy goat. The DPP and milk samples were analysed for its FA profile applying GC-FID and followed by chemometric techniques, namely, PCA and PLS. Results indicated that the n-6/n-3 ratio was the highest for the unsupplemented group compared to the DPP-treated goats with lower n-6/n-3 ratios. The M30 group showcased the most promising health-related class of FAs viewed by 3D PCA and PLS model clustering patterns, in particular monounsaturated FA (MUFA) (C18:1n9c or oleic acid). These results suggest that Mariami DPP supplementation at higher doses and time to lactating Saanen-Boer cross goats can be a means to milk FA quantity and quality enhancement and that chemometrics *via* pattern recognition can be useful statistical tools when dealing with overwhelming data.

## 1. Introduction

Sharifi et al. [1] reported that fatty acid (FA) quality and quantity in milk can be changed by diet especially by factors affecting the rumen fermentation, including the ratio of acetate (2C) to propionate (3C) concentration in the rumen fluid in goats. Nudda et al. [2] summarized several studies that utilized sunflower seed, palm, olive, soybean, canola, and castor oils in sheep on conjugated linoleic acid (LA), vaccenic acid, linoleic acid, and *α*-linolenic acid that resulted in varying levels of total FA (g/100 g) in the milk and cheese.

LA and linolenic acid are members of two well-known classes of PUFA: n-6 (omega-6) and n-3 (omega-3) series. The importance of the n-6/n-3 ratio has been suggested to decrease incidences of noncommunicable diseases (cardiovascular diseases, cancer, inflammatory, and autoimmune diseases) [3]. The unsaturated FAs are usually called “healthy fats,” referring to their impact on the cholesterol level in the blood [4, 5]. Additionally, PUFA reduces cholesterol levels stronger than MUFAs [6]. Moreover, oleic acid (C18:1 *cis*-9) and linolenic acid (C18:3 *cis*-9, *cis*-12, *cis*-15) which belong to the omega-3 family have anticancer and antiatherogenic properties [5, 7, 8]. Besides their effects on cholesterol levels, LA (C18:2 *cis*-9, *cis*-12) being the most vital in the omega-6 family improves the cell's sensitivity to insulin and thus reduces the incidence of type 2 diabetes [9]. Previous dietary recommendations propose a dietary n-6/n-3 ratio lower than 5 to reduce the risk of cardiovascular diseases, cancer, autoimmune disorders, allergies, obesity, and some mental disorders [10].

Afiq et al. [11] and Hossain et al. [12] stated that date pits (DP) are a potential source of FAs. Al-Shahib and Marshall [13] analysed 14 varieties of date pits including Sukkary, Safawy, Sofry, Anabarah, and Rabeeah among others and reported that these different varieties of date pits exhibited different FAs with varying percentages. Furthermore, Nehdi et al. [14] noted that the oleic acid and linoleic acid contents in the date pit oil are 50% and 19% of the total FA composition, respectively. Additionally, date pits are often softened *via* soaking in water to feed camels, sheep, goats, and horses or crushed dry and mixed with chicken feed [15]. In general, El Hadrami and Al-Khayri [15] also reviewed and summarized the socioeconomic impact of dates and date pits.

Martinez Marin et al. [16] observed that coupling with linear discriminant analysis (LDA), which is a chemometric tool, could effectively predict the type of diet consumed by dairy goats through assessing the milk FA contents. Moreover, Bassbasi et al. [17] quantified the solid nonfat (SNF) in raw milk samples using Fourier Transform Infrared (FTIR) and applied chemometrics techniques such as partial least squares (PLS). Chemometrics are extensively applied to find common variation patterns in complex data [18].

While milk fat has a greater sensitivity to dietary influence [19] plus the economic value that the DP as an agricultural waste byproducts carry, it is hypothesized that the quality of milk FA could be affected by the DP supplementation as DP have nutritional qualities suitable for animal consumption and are available as waste byproducts throughout the year in tons. Hence, the purpose of this work is to identify and quantify FA profiles and to determine the n-6/n-3 FA ratio in milk from early lactating goats supplemented with Ajwa DPP (highly valued dates) and Mariami DPP (agricultural waste byproduct) at various doses and time, applying chemometrics.

## 2. Material and Methods

### 2.1. Goat Management

Ethical approval for the animal study was approved by the Animal Ethics Committee (AEC) of Universiti Sains Islam Malaysia (USIM/AEC/AUP/2016 (3)]) Thcin Sg. Buloh, Selangor, Malaysia (GPS: 3.197592; 101.524848). A total of six groups of one year old Saanen-Boer cross female goats (*n* = 4per group) and a mean body weights of24.89 ± 3.08 kg were applied in the study. The goats were first subjected to a general health inspection by the farm owner and the veterinarian in the research team to ensure that the goats were in general good health. The goats were then adapted to the research setting and fed with a basal diet, comprised of several feed types as in Table 1 prior to kidding. All goats were randomly divided into two goats per cubicle (6  × 5 feet) and left for natural impregnation over a month's period. Normal gestation period was about 5-6 months, and the kids were allowed to suckle for the first week, and after then, they were separated from their mothers. The lactating goats were then sorted appropriately to ensure that they were healthy throughout the study period [20].

### 2.2. Chemicals

Chloroform (CHCl_3_), sulfuric acid (H_2_SO_4_), and anhydrous sodium sulphate (Na_2_SO_4_) were obtained from Merck (Germany), and methanol (CH_3_OH) and sodium hydroxide (NaOH) were procured from Fisher Chemicals (UK). The 37 FAME standard mix was purchased from Supelco (USA). All reagents, solvents, and standards were of analytical grade.

### 2.3. Instrumentation

Gas chromatography (GC) analyses were performed on an Agilent 7890A GC equipped with a split capillary (1 : 50) injector quantified by a flame ion detector (FID). Analytical separation was achieved on HP-88 capillary column (100 m × 0.25 mm) with 0.20 *μ*m film thickness. Helium was used as the carrier gas (with a constant flow rate of 1.9 mL/min). The air, hydrogen (H_2_), and auxiliary nitrogen (N_2_) gas pressures for the detector were 400 mL/min, 30 mL/min, and 30 mL/min, respectively. The temperature settings were as follows: injection port at 270°C and FID temperature at 280°C. The oven temperature was held at 100°C for 1 min followed by 120°C for 1 min, programmed to 175°C at the 5°C/min, and held for 10 min; then programmed to 210°C at 5°C/min and held for 5 min; and then programmed to 230°C at 5°C/min and held for 7 min.

### 2.4. Basal Diet (BD) and Date Pit Powder (DPP) Supplementation

Each lactating goat was given a BD inclusive of approximately 500 g each of pellet, fresh Napier leaves, and rice hay, respectively, per day throughout the experimental trial (Table 1). Ground DPP from two cultivars, Ajwa (highly valued) and Mariami (byproduct from the production of confectionaries) that were applied in this research, were purchased from Syarikat Abdul Gaffar (SAG), Penang, Malaysian.

### 2.5. Feeding Trial

Pellets from Nutri Vet Trading Company, fresh Napier leaves, and rice hay were administrated to the goats separately at 8 : 00 a.m., 12 : 00 noon, and 4 : 00 p.m. daily, respectively. The daily dose of 10 g of Ajwa DPP (A10), 20 g of Ajwa DPP (A20), 10 g of Mariami DPP (M10), 20 g of Mariami DPP (M20), and 30 g of Mariami DPP (M30) per goat per day was fed to the goats by mixing with the pellets, while goats in the control group were only fed with the BD. Postmorning meal session, the feeding tray was empty assuming all DPP was ingested along with the pellet. The percentage of DPP in relation to the BD is shown in Table 2. Clean water was supplied by an automated dispenser *ad libitum*. The trial began on the eighth day of lactation and lasted for 12 weeks.

### 2.6. Sample Collection and Preparation

Milk collection was done manually. The udders of the goats were dabbed with a clean damped cloth prior to hand-milking once daily into individual labelled newly manufactured milk plastic bottles until the udder was reasonably empty at 2 : 00 p.m. by an experienced farm worker who was supervised by the owner. The milk samples were then immediately stored in the chiller at 2-4°C. Subsequently, the kid was left to suckle until the next day [22] before being separated from its mother again, prior to the morning meal (8 : 00 a.m.) until the milk sampling time (2 : 00 p.m.) of the same day. The milk samples were then brought in an ice box to the laboratory facility twice a week, volume-measured and were pooled after each visit, frozen in 50 mL Falcon bottles, freeze-dried, and stored in powdered form at -20°C for further analyses.

### 2.7. Determination of FA Profile Using Gas Chromatography Flame Ionization Detector (GC-FID) Spectrometry

A one-step FAME analysis by Indarti et al. [23] was performed with some modifications. Monthly (end of months 1, 2, and 3) freeze-dried milk samples (100 mg) were weighed and transferred into 10 mL screw-top glass bottles. Acidified MeOH at a ratio of 85% MeOH to 15% H_2_SO_4_ was prepared by slowly adding MeOH to acid and mixed to avoid rapid interaction. Then, MeOH: H_2_SO_4_ mixtures (2 mL) followed by CHCl_3_ (2 mL) were carefully added to the sample. Nitrogen (N_2_) gas was introduced to the mixture for 15 s, and the bottles were tightly capped and vortexed for 2 min. Next, the bottles were placed on the heater block and heated at 80°C for 30 min. The samples were then left to cool at room temperature followed by the addition of distilled water (1 ml), vortexed for 30 s, and left to stand for overnight to allow the formation of two layers. Using a micropipette, the lower layer was carefully transferred into an Eppendorf tube. Small amounts of anhydrous Na_2_SO_4_ were added to precipitate the nonderivatized components. The samples were then filtered using nylon syringe filter (0.22 *μ*m) to remove the precipitate that otherwise could clog the column. The prepared FAME samples were kept in the freezer (-20°C) until GC-FID analysis.

The FA profile of DPP (100 mg) was similarly prepared as described above. Sample size injected for each analysis was 1 *μ*L. DPP and goat milk FAs were identified by comparing the retention times of FAME with the standard mixture of 37 FA components.

### 2.8. Statistical Analyses

Data were analysed using the SPSS statistical package, IBM version 20 (SPSS Inc., Chicago, IL), and were expressed as mean ± SD. The differences between the means of the treatments were compared at a significance level of *p* < 0.05 using ANOVA and post hoc comparisons applying Duncan's test and a mixed model procedure. Repeated measurements and the random effect of the goats were also considered. Analysis was carried out to determine the effect of diet (fixed effect) on FA composition as dependent variables. Chemometrics was then applied using PCA for potential cluster analyses and followed with PLS for prediction purposes *via* Unscrambler X (Camo Software, Oslo, Norway). Data were maximum-normalized before the analysis.

## 3. Results and Discussion

### 3.1. FA Profile in DPP

The method of Indarti et al. [23] to synthesis FAME from the samples was applied in this study but with modifications, and due to that, their results showed that the direct FAME synthesis method using methanolic sulfuric acid reagent is an alternative and more suitable method (95% total FA recovery) for the preparation of FAME from fish oil and cod liver oil compared with the conventional method (80% total FA recovery).

DPP FA profile analysis was done to ascertain whether similar DPP FAs would be influential and correlated to the FA in milk and *vice versa*. The mean percentages of individual FA detected in both Ajwa and Mariami DPP are presented in Table 3. Oleic acid (C18:1 *cis-*9) showed the highest concentration of 44.87 and 46.62% in Ajwa and Mariami DPP, respectively, whereas linoleic acid (C18:2 *cis-*6) was detected in the least concentration with 7.88 and 7.29%, correspondingly. The carbon chain length ranged from C12:0 to C18:0 for both date pit cultivars. Mariami DPP is an agricultural waste byproduct and is available yearly in tons compared to Ajwa DPP, which is expensive (can reach 3 times the price of the next best variety), belonging to the holy city of Al Madinah Al Munawara and its adjoining areas in Saudi Arabia [24].

Similar FAs with the highest percentages that were observed in Table 3 were also listed by Sawaya et al. [24], whereby DP FA displayed that oleic acid (52.2%), lauric acid (24.2%), myristic acid (9.3%), palmitic acid (9.9%), and linoleic acid (8.5%) were the bulk of the total FAs. In addition, Devshony et al. [25] demonstrated that the most abundant FAs in DPs were oleic acid (41-44%) and lauric acid (19-24%), followed by myristic acid, palmitic acid, and linoleic acid (8-15%). Hossain et al. [12] showed that the FA compositions in various DP species varied from C10:0 to C18:0 chains compared to this current finding. Oleic acid, which is categorized as long-chain FA (LCFA) when taken in the diet, will increase the high-density lipoprotein (HDL) content and, at the same time, lowers the low-density lipoprotein (LDL) content in blood [26]. Devshony et al. [25] and Besbes et al. [27] stated that the main SFA and unsaturated FA (UFA) of DP are lauric acid and oleic acid, respectively. In contrast, stearic, capric, and caprylic acids are present in minor amounts [28]. Other additional FAs in DP include margaric, arachidic, behenic, palmitoleic, and linolenic acids [29].

Apart from the powder form, Devshony et al. [25] proved that FA can also be extracted from DP oil. The researchers suggested that DP oil may be regarded as oleic-lauric oil, distinct from palm oil (palmitic-oleic) and palm kernel/coconut oils (lauric-myristic). In general, DP oil would appear to be useful in cosmetics, pharmaceuticals, soaps and detergents, chemical intermediates, and food applications [25]. However, DP oil showed low content of linoleic acid compared to the commonly consumed vegetable oils and a lower degree of unsaturation [24].

### 3.2. FA Profile in Milk from Goats Supplemented with DPP

Since published works on FA analysis of milk from dairy goats receiving DPP supplementation and subsequent analysis using chemometrics are scarce, discussion pertaining to the results that were generated from this study was compared to those studies which touched quite similar issues raised by this research.

The benefit of using GC instead of mid-infrared spectrometry is that it is a more accurate measurement of FAs but with lower concentrations detected in bovine milk fat [30]. Despite using GC-FID, Andreotti et al. [31] determined the FA composition in goat milk applying ^13^C NMR.

The number of goats used in this study for each treatment was *n* = 4, but for the analyses *per se*, only three data from three respective goats was considered. This was similar to the work done by Martinez Marin et al. [32] who utilized three animals per treatment during 15 days. According to few studies by Chilliard et al. [33] and Fievez et al. [34], this time would be sufficient to get the response of FA in milk fat similarly to those obtained with extended periods. Nevertheless, the changes in goat milk FA composition and concentrations after DPP supplementation at the respective months were seen in Tables 4–6.

In spite of the comparable individual SFA percentages found in the Ajwa and Mariami DPP (C12:0, C14:0, and C16:0), it did not affect the same FAs in the milk as there were no profound trends in the individual milk samples that were obvious. This was also the result of a low percentage of FAs in the DPP. Effects were only seen in the A20 group for C12:0 (lauric acid). Although the content is lower than in Mariami DPP, supplementation of Ajwa DPP at higher doses (20 g) could affect milk FA content. Nevertheless, SFA (C4:0, C11:0, C12:0, C14:0, and C15:0) in the control group showed a significant (*p* < 0.05) increase compared to the rest of the total SFA contents. Similarly, A20 exhibited the same number of significant individual SFA with C4:0, C6:0, C8:0, C10:0, and C12:0 being increased as in the control group (Table 4). On the contrary, goats supplemented with Mariami DPP exhibited lesser SFA content as an indicator for the prevention of cardiovascular problems [35]. A similar outcome was also seen in the works of Morsy et al. [36],who proved that milk from goats which received sunflower seed (SS) or sunflower oil (SO) supplementation displayed a decreased in SFA content. However, Osmari et al. [37] did an implication summary of FA in cardiovascular heart disease (CHD) development, stating that stearate and short-chain (C7:0–C11:0) SFA do not raise serum cholesterol, therefore not influencing the cholesterol concentration in the blood.

Apart from that, Adeyemi et al. [38] used oils of a blend of canola oil and palm oil (BCPO) at different percentages in parallel to the feed of Boer bucks to assess FAs but in the ruminal fluid and *triceps brachii* muscle. Their results emphasized that the ruminal concentrations of C18:0, n-3 FA, and total FA increased significantly (*p* < 0.05), while C12:0, C14:0, C15:0, and n-6 FA decreased with increasing BCPO. Analysis of *triceps brachii* muscle showed that the concentrations of C16:0, C14:0, and C18:2 n-6 were lowered sigificantly (*p* < 0.05), while C18:1 n-9, C18:3 n-3, and C20:5 n-3 were higher in oil-fed goats compared with control.

As milk yield could be affected by the seasonality of pasture, the diet type could also modify the milk FA. A higher level of unsaturated FA (UFA) in the animals' diet can increase the desirable FA (DFA) in milk [37]. Although it is shown that supplementing DPP which has both oleic acid (C18:1 *cis*-9) and linoleic acid (C18:2 *cis*-6) as UFA to the lactating goats, it did not affect the same FA in the milk. However, only oleic acid was found in the milk samples but with no significant effect despite being the bulk (~50%) of the FA in the DPP. In addition, it has been shown that the breed also affects FA in the milk of dairy goats [39], but there is non-yet known interaction between the Boer cross breed and nutritional factors [37].

The differences in milk FA composition observed in this study compared to others also depended on many other factors; an example is the negative energy balance (NEB). In periparturient cows, the change in FA composition in milk and blood may be due to NEB, whereby it is a situation when energy deficit occurs in high-producing dairy cows, which are rendered from energy intake capacity during the early lactation period thereby not meeting the energy requirements for milk yield and maintenance [40]. Nonetheless, the most important factor among them is actually diet management as stated by Min et al. [19].

A study by Al-Suwaiegh [41] indicated that the fat percent and yield were not significantly (*p* > 0.05) different among all his DPP-treated animals, which hypothesized that goats fed with diets containing DPP produce an adequate amount of acetate required for milk fat synthesis. In another perspective, although lipid supplementation to lactating goats' diets typically enhances milk fat percentage and yield, thereby distinguishing goats from cows, the response in terms of milk FA composition is close to that observed in cows [42].

Nudda et al. [2] and Chilliard and Ferlay [43] reported that the *de novo* synthesis of FA in milk originated from acetate and *β*-hydroxybutyrate produced by rumen fermentation. These volatile FAs (VFA) are the main carbon sources for the secretory cells of the mammary gland involved in the *de novo* synthesis of short-chain FA (SCFA) (C4:0-C14:0) and a portion of C16:0. The remaining part of C16:0 and almost all LCFA (C18:0-C22:0) in milk come from the lipids circulating in the blood, originated from absorption in the small intestine or mobilization of adipose tissue. Though, acetate may also indicate the incidence of elevated somatic cell count (SCC) or mastitis [44].

In addition, Sharifi et al. [1] emphasized that the total VFA concentrations (acetate, propionate, butyrate, valerate, isovalerate, and acetate: propionate) of the rumen fluid were not significantly (*p* > 0.05) affected by low-quality date palm (LDP) treatment. However, the increased dietary LDP content raised the ruminal molar proportions of propionate (*p* < 0.05) and valerate (*p* < 0.05) and, at the same time, decreased the ruminal molar proportions of acetic acid and acetate: propionate ratio (*p* < 0.01). Alternatively, small FA carbon molecules could be produced by the microbial population of the rumen [7], which degrade and ferment dietary carbohydrates and proteins to produce VFA. The most important of these molecules are acetate, propionate, and butyrate because acetate and butyrate are precursors of milk SCFA and medium-chain FA (MCFA). Propionate, on the other hand, may also be indirectly involved in the synthesis of some branched-chain FAs through the incorporation of methylmalonyl-CoA, its carboxylation product [45]. Additionally, Nudda et al. [2] showed that the content and source of neutral detergent fiber (NDF) and nonfibrous carbohydrates (NFC; i.e., sugars, starch, and soluble fiber) in the diet influence VFA profile in the rumen of sheep.

MUFAs (C17:1, C18:1 *cis*-9, and C20:1) in the M30 group were significantly (*p* < 0.05) increased when paralleled to the other DPP-treated goats (Table 5). In general, all Mariami groups exhibited significant (*p* < 0.05) improved mean levels of MUFA when compared to the other groups. Meanwhile, most of the detected milk PUFA was significantly different at *p* < 0.05 for the majority of the Mariami groups (Table 5) with C18:2 *cis*-6 being significantly (*p* < 0.05) different and was comparable to MUFA. With the notion that normal progress of lactation would result in highly favourable changes, which is the increased in MUFA and PUFA content, and SFA decrease [46], Morsy et al. [36] clarified that PUFA are not synthesized by ruminants; thus, their concentration in milk depends on the amount of PUFA absorbed from the intestines. Kishino et al. [47], however, indicated that FAs are generated through PUFA metabolism of gastrointestinal microorganisms. Their findings suggested that lipid metabolism by gastrointestinal microbes affects the health of the host by modifying FA composition. In another perspective, Silanikove et al. [48] reported several studies that showed that milk and cheese from grazing goats had better quality parameters for human nutrition than that produced from the milk of goats fed indoors, with concentrations of conjugated linolenic acid and PUFA being higher in the milk fat of pasture-fed goats, together with a significant reduction in the atherogenic index of the FA. By using the semi-intensive protocol as shown by those researchers, a different result may arise from the results generated in this study, as this current study practiced the fully intensive goat management procedure.

Martinez Marin et al. [32] detected the majority of standard FAs in goat milk using GC compared to [49] who only found 22 FAs. These were more than the FAs detected in this present study. All of which utilized the 100 m length column but applied different extraction protocols and programmed temperature methods. In the local context of Malaysia, SydJaafar et al. [21] (unpublished work) also analysed goat milk FA and among them were milk from the same goat species as in this experiment, applying similar protocols. They found 24 FA considering the milk samples that were analysed in liquid form compared to this present study which analysed the milk samples in powdered form.

#### 3.2.1. Milk FA Length Classification

DPP supplementation was seen to significantly (*p* < 0.05) affect milk FA chain length with A20 showing significant (*p* < 0.05) effects on SCFA; A20 and control on MCFA and M30 on LCFA. Furthermore, there was an increasing trend for SCFA throughout the 3 months in the control and M20 groups in contrast to A20 which presented a decreasing trend. Although MCFA increment and decrements were not direct throughout the study duration, it showed a relative increase for all Mariami DPP groups (M10, M20, and M30) but with a decrease for the remaining groups (control, A10, and A20).

As seen in Table 6, A20 treatment resulted in ~25% of the significant (*p* < 0.05) increased FA being classified as SCFA. This was not the case for the control and Mariami DPP-treated groups. Jenness [50] reported that almost 20% of the FAs of goat milk are grouped into the SCFA category (C4:0 to C12:0) compared to l0-20% for cow milk. Lipases attack the ester linkages of the SCFA more readily, so these differences may contribute to more rapid digestion of goat milk fat. Similarly, Silanikove et al. [48] and Haenlein [51] specified that goat milk contains a higher percentage of SCFA and MCFA, facilitating degradation by lipases, and has lower levels of *α*_S1_-casein leading to the generation of a less compact and more digestible curd compared to cow milk. Among the SCFA, butyrate is an important flavor component of milk [52]. In spite of that, Mariami DPP group exhibit reductions in the trend for the LCFA (Table 6). A study by Osmari et al. [37] indicated that MCFA was significantly (*p* < 0.05) altered compared to LCFA due to supplementation with either three roughages (sorghum silage, maize silage, or mulberry hay) to Saanen-Boer crossed (same species used in this current experiment) goats.

#### 3.2.2. Milk FA Degree of Saturation

Mariami DPP-treated groups have shown an increase in total milk SFA (TSFA) including the control compared to the Ajwa DPP group, although significant (*p* < 0.05) differences were only seen in the control (Table 6). In the case of MUFA, the opposite scenario was seen for all groups when compared to the TSFA, with Mariami DPP and control groups that were in decreasing trend, and Ajwa DPP group in increasing trend. Significant (*p* < 0.05) differences were only attributed to the M30 group. While there was no significant (*p* > 0.05) difference in total PUFA (TPUFA) among all groups, all DPP-treated groups exhibited a higher mean of PUFA compared to the control (Table 6). In contrast, the results shown by Chilliard and Ferlay [43] revealed that feeding goats with high or low forage with different concentrations of added linseed oil, vitamin E, or extruded linseed supplementation and different ratios of either linseed oil or extruded linseed supplementation strongly decreased the desaturation ratio and simultaneously increased linolenic acid and *trans*-FA percentages in goat milk.

Another reason for the increment of unsaturated FA (UFA) levels is due to the increase of energy needs at the onset of lactation for example, in cows where it is associated with mobilization of body reserves. As a result of extended fat breakdown (lipolysis) of adipose tissue, there is a partitioning of nonessential FAs (NEFAs) into the bloodstream. These events will influence the FA profile in milk for example, by an increase in UFA concentration, mainly C18:1 [53, 54]. Thus, the FA level is an indicator of the process of lipolysis in milk. Furthermore, during the peak of lactation, even 30-40% of the body fat may be subjected to lipolysis, which causes changes in the milk FA contents [46]. The same trend of milk fat composition was observed with a higher concentration of UFA in the milk of cows during NEB [55]. On the contrary, the presence of UFA has been related to inflammatory diseases such as mastitis and metritis in cows [56]. In humans, an increased level of circulating NEFAs in the blood is associated with increased systemic inflammatory conditions [57]. From the food perspective, the increase in FA contents due to lipolysis affects the aroma and thereby the quality of fresh and processed products [58].

Interestingly, goat milk exceeds cow milk in MUFA, PUFA, and medium-chain triglycerides (MCT), which all are known to be beneficial for human health, especially for cardiovascular conditions [51]. Dewhurst et al. [59] recommended that total fat, SFA, n-6 PUFA, n-3 PUFA, and trans-FA should contribute <0.15–0.30, <0.10, <0.05–0.08, <0.01–0.02, and<0.01 of total energy intake, respectively. In addition, a diet with microalga supplementation significantly increased the concentration of n-3 FAs such as *α*-linolenic acid, eicosatrienoic acid, and docosahexaenoic acid in goat milk with 1.35% vs. the control (1.02%) [60]. Moreover, Morsy et al. [36] added a daily dose of sunflower seed (50 g/h/d) or sunflower oil (20 ml/h/d) in the diets of lactating Damascus goats and proved that it decreased TSFA but increased total CLA and total UFA in milk with more beneficial effects for SO than SS. In a separate study by Kholif et al. [61], the profiles of FAs were significantly affected by both dietary forage and cattle species whereby cow milk contained significantly higher contents of *cis*-9, *trans*-11, and 18: 2 CLA (0.59 g/100 g milk fat) than that in buffalo milk (0.47 g/100 g milk fat).

### 3.3. Omega-6: Omega-3 FA Ratio in Milk

After a month of DPP supplementation, the initial level of goat milk n-6/n-3 ratio varied among all the groups except for M10 and M30 that exhibited an increasing trend and the control with the significant (*p* < 0.05) highest value. However, with extended time, the control had their n-6/n-3 values decreased. A10 showed the least n-6/n-3 FA ratio mean when compared to the other groups. These data provide an overview of the n-6/n-3 FA ratio, which leads to a lipid index for general health with a value as much as possibly close to 1 are considered protective against degenerative pathologies and that a very high n-6/n-3 FA ratio is considered detrimental for human health [3]. Simopoulos [62] and Simopoulos [63] reviewed several clinical intervention studies which support the view that decreasing the n-6/n-3 FA ratio results in an increased protection against degenerative diseases.

Morsy et al. [36] displayed that both sunflower oil and sunflower seed additives decreased TSFA and increased both total CLA and UFA in milk with more beneficial effects for SO than SS, which then reduces the n-6/n-3 ratio. Osmari et al. [37] studied the FA ratio in milk produced by Saanen-Boer cross goats in influencing the milk FA but used sorghum silage or mulberry hay as part of the animals' diet. They found out that supplementing the goats with either of the two feeds gave better milk lipid index (atherogenic and thrombogenic indices), which may help to prevent coronary disease. Despite that, a great influence in human diet and lifestyle was observed due to the agricultural revolution, which introduced cereals and grains high in n-6 FA in the diet. Over time, the human population experienced a dramatic increase in the consumption of vegetable-based seed oil rich in n-6 FAs and a decrease in n-3 FA intake. This resulted in the increase in the n-6/n-3 FA ratio, which was obvious in the Western diet which ranged between 15 : 1 and 20 : 1 [62, 63].

### 3.4. Chemometrics

#### 3.4.1. Principal Component Analysis (PCA)

PCA and PLS-DA (or PLS) are primarily classification methods, exploring class differences and focusing on explanatory metabolites [64], and in this study, the focus on milk FA changes due to DPP supplementation. There were no discrete cluster differences among the DPP-supplemented groups (figure not shown). For the majority of PCA analyses done in other studies, there is no 100% clustering of different groups due to the samples, which are of biological origin and thus are exposed to confounding factors that are uncontrollable [65]. However, Arifah et al. [66] showed the opposite when comparing between goat and horse milk using FTIR spectroscopy data visualised using Cooman's plot. Additionally, Blasko et al. [67] developed a chemometric deconvolution procedure that allowed the determination of the contents of the studied CLA isomers in ewe and cow milks. Determined contents of CLA isomers allowed differentiation of the milk from ewes fed on pasture compared to those who were fed with total mixture rations. The same researchers also differentiated between summer and winter cow milks using the CLA isomers.

**Figure 1:**
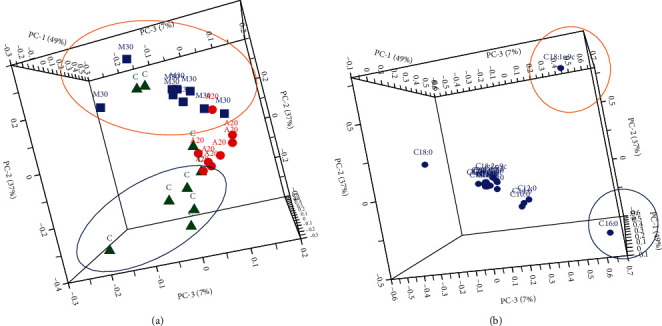
3D PCA of milk FA comparison among the control and highest significant (*p* < 0.05) milk-yielding groups. (a) Score plot: green triangle = control; red circle = 20 g Ajwa DPP; blue squares = 30 g Mariami DPP. (b) Loading plot: individual FAs.

On the contrary, possible clusterings were obvious among the three groups separating between the control and the significantly (*p* < 0.05) highest milk-yielding groups (A20 and M30) (results from a separate study) in a 3D PCA illustration as shown in Figure 1. This 3D view was generated when the 2D diagram did not show a clear separation of the groups. The analysis was done to ascertain whether with a significant milk yield increase, would there also be differences in milk FA profiles as well. This envisages possible milk quantity and FA quality identifiers due to DPP treatment. It manifested that the significant highest milk-yielding groups (A20 and M30) also presented with different qualities and quantities of FA with C18:1 *cis*-9 that was linked to the M30 group, whereas A20 had all other common FAs among the control and M30 groups. The milk from the control group, however, was associated to C16:0 and was in agreement with Myrzakozha et al. [68] whereby the authors exposed that C16:0 was comparable among camels, cows, goats, and mares with cows and goats exhibiting the highest contents. Meanwhile, Zhang et al. [49] indicated that the goat milk yield was not affected by neither a high-quality roughage containing Chinese wild rye hay, corn silage, alfalfa, and concentrate nor by a low-quality roughage comprising of Chinese wild rye hay, corn stover, and concentrate treatment with milk FA composition not being different between the treatments, except for C18:3 n-3 (0.27 vs. 0.15 g/100 g), respectively.

**Figure 2:**
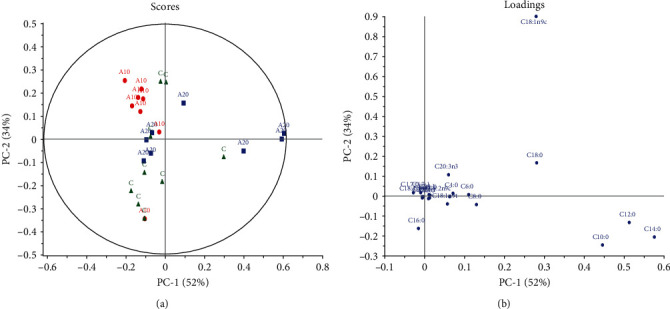
PCA of Ajwa DPP groups vs. control. (a) Score plot: green triangles = control; red circles = 10 g Ajwa DPP; blue squares = 20 g Ajwa DPP. (b) Loading plot: individual FAs.

**Figure 3:**
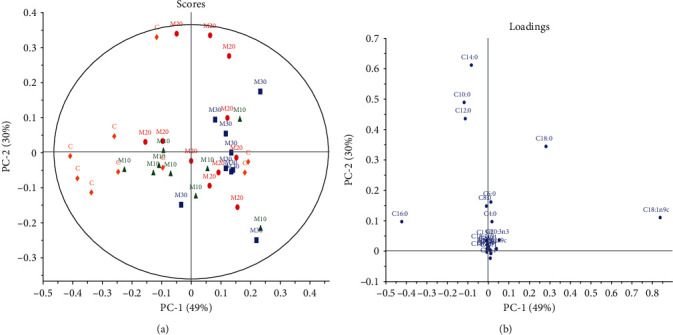
PCA of Mariami DPP groups vs. control. (a) Score plot: yellow diamond = control; green triangles = 10 g Mariami DPP; red circles = 20 g Mariami DPP; blue squares = 30 g Mariami DPP. (b) Loading plot: individual FAs.

When the comparison *via* PCA was made between the control and Ajwa DPP groups (Figure 2), a considerable separation between the groups indicating differences in FA concentrations was seen for MUFA with C18:1 *cis*-9 being increased in the majority of the Ajwa DPP groups. This was a better plot compared to the PCA model generated by the control and Mariami DPP groups (Figure 3). Figure 2 indicates that Ajwa DPP dose had some effect on milk FA quality as A10 was shown to have a separate cluster than A20. The same scenario which observed Mariami DPP group that had an increase in C18:1 *cis*-9 when compared to the control was further emphasized. Moreover, Myrzakozha et al. [68] showed that camel milk fat had the largest proportion of C18:1 *cis*-9 and was subsequently to goats, cows, and mares.

The control group was homogenously located in the leſt quadrant of the score plot and associated with increased SFA (C16:0) levels, while both Ajwa DPP and Mariami DPP groups were characterized by a more heterogeneous distribution as observed in Figures 1 and 2, respectively. PC 1 explained 52% of the data variation in Figure 2 compared to PC 2 which only explained 34%. Meanwhile, PC 1 and PC 2 accounted for 49% and 30%, respectively, in Figure 3. Caboni et al. [69] revealed that SFA levels were higher in high somatic cell count (SCC), particularly the short- and MCFA (C8:0, C10:0, C12:0, and C14:0), while long-chain (C16:0 and C18:0) SFA together with C18:1-9cis accounted for lower percentage values. Besides PCA, Martinez Marin et al. [16] presented that from 84 variables, linear discriminant analysis (LDA) permitted the identification of 20 variables as useful predictors. The LDA is a convenient method to classify milk fat samples according to the certain vegetable oil added to a basal diet from several FAs quantified in milk fat.

It was noted that C14:0 and C18:1 *cis*-9 were interchangeable in position for both Figures 2 and 3 even though both figures did not compare the same samples except for the control. It is seemingly that two control samples had contributed to the C18:1 *cis*-9 to the PCA. The same applied to C14:0, whereby another control sample also had contributed to the change in both the corresponding score and loading plots. Moreover, despite the fact that the two DPPs were of the same species (*Phoenix dactylifera* L.), Ajwa and Mariami DPP supplementation did show an effect on milk FA quality and quantity when compared to each other. In Figure 3, Mariami DPP group had the advantage of elevated C18:1 *cis*-9 concentrations when paralleled to the Ajwa DPP group. Moreover, C16:0 was elevated in the Ajwa group compared to the Mariami group. However, both groups were heterogeneously distributed.

#### 3.4.2. Partial Least Square (PLS)

When PCA did not reveal discrete clustering among the groups, a supervised PLS regression method may be performed. The test evaluates the repeated variables between groups and highlights the differences between groups as previously used in, for example, other metabolomics approaches. It can be used to model and predict milk FA contents in the DPP-supplemented goats. The fact that PLS searches for the factor subspace most congruent to both dependent and independent variables, its predictions are usually better than using other multilinear regression methods, especially when a large number of collinear variables are present as independent variables [17]. Wold et al. [70] stated that PLS is a multivariate calibration method where both the independent and dependent variables are related using regression in which overfit is probable.

**Figure 4:**
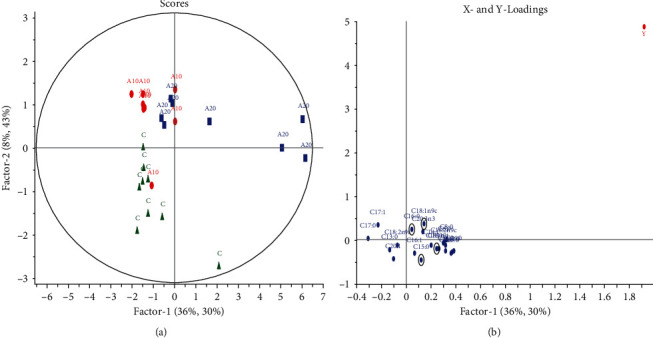
PLS analysis of Ajwa DPP groups vs. control. (a) Score plot: green triangles = control; red circles = 10 g Ajwa DPP; blue squares = 20 g Ajwa DPP. (b) Loading plot: individual FAs.

**Figure 5:**
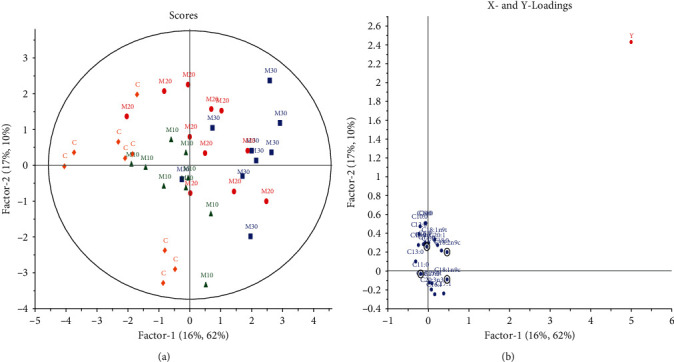
PLS analysis of Mariami DPP groups vs. control. (a) Score plot: yellow diamonds = control; green triangles =10 g Mariami DPP; red circles = 20 g Mariami DPP; blue squares = 30 g. (b) Loading plot: individual FAs.

PLS analysis indicated the potential use of both Ajwa DPP and Mariami DPP in the prediction models as illustrated in Figures 4 and 5, respectively. The scattering of samples in the PLS-derived score plot was dissimilar from that of the PCA. Investigation of the factor 2 of the PLS loading plot showed the FA which strongly contributed to the separation of the FA profiles based on the different Ajwa DPP doses (Figure 4). Ajwa DPP groups were placed on the positive quadrant of PLS factor 2, whereas the control was on the negative quadrant with C15:0, C16:0, C18:0, and C18:1 *cis*-9 that were significant in contributing to the PLS model as summarized in Table 7. Likewise, Figure 5 depicts the further separation of higher Mariami DPP doses away from the control but having a lesser clustering effect among the various doses. When *Q*^2^ value lower than 0.5 is attained (as seen for PLS model of A vs. C), SIMCA users should verify that the quality parameters are constant towards permutation of the rows in their dataset [71]. Triba et al. [71] also highlighted that a large discrepancy between *R*^2^ and *Q*^2^ indicates an overfitting of the model through the inclusion of too many components as depicted in model of A vs. C. Nonetheless, with extended DPP doses either for both Ajwa and Mariami, it is envisaged that FA prediction can still be done. Accordingly, Bai et al. [72] reported that the FA profile can be a potential tool for validation of organic milk's authenticity in Inner Mongolia of China. The contents of FA in milk are related to feed, season, animal breed, processing, and storage. They applied PCA in addition of PLS-DA to PLS compared to this study.

## 4. Conclusions

Varying goat milk FA profiles were successfully established due to different DPP supplementation strategies, with M30 exhibiting the most promising health-related classes of FAs. This was in spite that FAs detected were lesser than in some other goat milk-based studies. This was mainly due to the different extraction protocols, programmed temperature methods, and mainly diet as discussed earlier. Nevertheless, n-6/n-3 ratio for all the treatments showed that the highest being the unsupplemented group and alternatively the DPP-treated goats had lower n-6/n-3 ratios which have potential health-related benefits especially to the cardiovascular system as stated by other published references. PCA highlighted the dissimilar groupings of various DPP treatments on individual FAs that indicated the effects of DPP on the milk samples. Subsequent PLS analysis showed that Mariami DPP gave a better prediction model based on the *Q*^2^ value (0.525) which was higher than the Ajwa DPP (0.267), however with a lower *R*^2^ (0.829) compared to *R*^2^ value of Ajwa (0.848). All in all, these data suggest that Mariami DPP as an agricultural waste byproduct can be an alternative supplement for goat milk FA quality enhancement and prediction and that chemometrics may assist in visualizing the effects of supplementation.

## Figures and Tables

**Table 1:** Nutrient composition (%) of the BD used in the feeding trial.

Feed type	Composition (%)				
	Dry matter	Crude protein	Fat content	Crude fiber	Total ash
Fresh Napier grass	15.18 ± 0.03	16.40 ± 1.34	1.97 ± 0.42	32.05 ± 0.68	12.26 ± 0.67
Grower pellet	90.57 ± 0.06	17.81 ± 0.88	5.15 ± 1.24	23.92 ± 1.72	7.90 ± 0.22
Rice hay	86.20 ± 0.12	6.19 ± 0.16	0.90 ± 0.24	37.38 ± 0.49	11.83 ± 0.20

Note: data = means of triplicate. Source: reproduced with permission from SydJaafar et al. [21].

**Table 2:** Feeding treatment comprising of BD and DPP supplementations.

Group	Treatments	BD + percentage (%) of DPP in diet
C	Control (untreated)	BD only
A10	10 g of Ajwa DPP	BD + 0.67 DPP
A20	20 g of Ajwa DPP	BD + 1.33 DPP
M10	10 g of Mariami DPP	BD + 0.67 DPP
M20	20 g of Mariami DPP	BD + 1.33 DPP
M30	30 g of Mariami DPP	BD + 2 DPP

Note: control = goats which did not receive DPP supplementation. *n* = 4; BD = basal diet; DPP = date pit powder.

**Table 3:** Mean percentage (%) of FA compositions in Ajwa and Mariami DPP.

No.	Fatty acids	Ajwa DPP	Mariami DPP
1	Lauric acid (C12:0)	21.70	22.46
2	Myristic acid (C14:0)	13.78	12.63
3	Palmitic acid (C16:0)	11.77	11.00
4	Oleic acid (C18:1 *cis-*9)	44.87	46.62
5	Linoleic acid (C18:2 *cis-*6)	7.88	7.29
Total		100.00	100.00

Note: DPP = date pit powder.

**Table 4:** Mean percentage (%) and colour-coding of SFA composition in goat milk from goats treated with DPP supplementation at their respective months.

	Treatments																	
	Control			Ajwa DPP						Mariami DPP								
Fatty acids	—			10 g			20 g			10 g			20 g			30 g		
	M1	M2	M3	M1	M2	M3	M1	M2	M3	M1	M2	M3	M1	M2	M3	M1	M2	M3
C4:0	2.80^∗^	2.34^∗^	2.49^∗^	2.24	2.18	2.27	2.38^∗^	2.57^∗^	2.52^∗^	2.01	2.38	1.89	2.58	2.39	2.55	2.66	2.60	2.19
C6:0	3.00	2.66	2.75	2.83	2.65	2.64	2.98^∗^	2.78^∗^	2.64^∗^	2.20	2.38	2.18	2.35	2.98	2.87	2.61	2.59	2.14
C8:0	2.81	2.70	2.56	2.88^∗^	2.80^∗^	2.71^∗^	3.33^∗^	2.91^∗^	2.59^∗^	2.11	2.41	2.33	2.21	2.91	2.68	2.70	2.24	2.59
C10:0	7.67	8.07	7.91	5.88	8.54	5.47	8.88^∗^	8.75^∗^	8.00^∗^	6.18	7.03	7.02	6.22	7.47	9.01	5.72	6.86	6.38
C11:0	0.07^∗^	0.12^∗^	0.21^∗^	0.07	0.14	0.12	0.17	0.13	0.10	0.09	0.10	0.08	0.12	0.06	0.11	0.06	0.06	0.07
C12:0	8.69^∗^	9.02^∗^	9.58^∗^	6.36	7.92	7.80	7.74^∗^	9.05^∗^	9.29^∗^	7.05	8.10	8.58	6.63	8.17	9.21	5.61	6.58	6.80
C13:0	0.04	0.04	0.03	0.01	0.03	0.00	0.02	0.00	0.04	0.04^∗^	0.03^∗^	0.07^∗^	0.01	0.02	0.00	0.01	0.01	0.01
C14:0	13.21^∗^	12.83^∗^	11.89^∗^	10.62	11.96	9.26	12.20	12.86	11.80	11.64	10.18	11.85	11.19	10.69	13.46	10.59	10.80	10.54
C15:0	0.47^∗^	0.68^∗^	0.55^∗^	0.45	0.55	0.41	0.45	0.35	0.22	0.54	0.38	0.53	0.55	0.37	0.50	0.32	0.37	0.44
C16:0	27.53	27.71	25.95	29.54^∗^	29.38^∗^	28.53^∗^	28.46	21.02	26.62	27.53	26.08	28.21	25.92	22.90	23.91	24.71	25.81	25.11
C17:0	0.40	0.41	0.54	0.54^∗^	0.51^∗^	0.67^∗^	0.40	0.21	0.29	0.53	0.15	0.54	0.71	0.45	0.37	0.33	0.53	0.45
C18:0	6.86	8.15	9.76	8.49	7.01	8.69	7.50	8.16	7.33	8.40	10.50	7.96	10.37^∗^	12.32^∗^	9.55^∗^	11.11^∗^	9.44^∗^	10.33^∗^

Note: M1 = month 1; M2 = month 2; M3 = month 3; Asterisks = significant (*p* < 0.05) differences in individual milk FA among the different treatments along the same row; green fonts = increasing trend throughout 3 months; red fonts = decreasing trend throughout 3 months; blue fonts = increasing trend; black fonts = decreasing trend; C4:0 = butyrate; C6:0 = caproate; C8:0 = caprylate; C10:0 = capriate; C11:0 = undecanoate; C12:0 = laurate; C13:0 = tridecanoate; C14:0 = myristate; C15:0 = pentadecanoate; C16:0 = palmitate; C17:0 = heptadecanoate; C18:0 = stearate.

**Table 5:** Mean percentage (%) and colour-coding of individual MUFA and PUFA compositions in goat milk from goats treated with DPP supplementation at their respective months.

	Treatments																	
	Control			Ajwa DPP						Mariami DPP								
Fatty acids	—			10 g			20 g			10 g			20 g			30 g		
	M1	M2	M3	M1	M2	M3	M1	M2	M3	M1	M2	M3	M1	M2	M3	M1	M2	M3
*MUFA*																		
C14:1	0.23	0.28	0.23	0.22	0.23	0.03	0.19^∗^	3.96^∗^	0.21^∗^	0.31	0.12	0.28	0.23	0.18	0.18	0.18	0.18	0.20
C16:1	0.78	0.81	0.75	0.56	0.42	0.82	0.54	0.53	0.69	1.14^∗^	0.93^∗^	1.09^∗^	1.00	0.60	0.76	0.90	0.98	1.10
C17:1	0.14	0.13	0.09	0.23	0.05	0.28	0.15	0.11	0.12	0.26	0.07	0.16	0.23	0.14	0.11	0.29^∗^	0.25^∗^	0.23^∗^
C18:1n9t	0.45	0.76	1.46	0.44	0.75	0.06	0.72	0.94	1.53	0.58	0.77	0.67	0.92	0.99	0.70	0.88	0.88	0.68
C18:1n9c	22.86	20.79	21.03	25.22	21.70	28.80	21.62	22.60	24.52	26.08	25.35	23.74	25.71	24.64	21.29	28.14^∗^	26.58^∗^	27.44^∗^
C20:1	0.26	0.40	0.03	0.25	0.26	0.04	0.09	0.00	0.11	0.23	0.17	0.31	0.43^∗^	0.37^∗^	0.19^∗^	0.49^∗^	0.40^∗^	0.42^∗^
*PUFA*																		
C18:2n6t	0.26	0.08	0.08	0.03	0.02	0.00	0.05	0.00	0.10	0.04	0.00	0.12	0.10	0.10	0.10	0.18^∗^	0.15^∗^	0.13^∗^
C18:2n6c	1.08	1.22	1.19	1.29	1.25	1.15	1.31	1.40	0.92	1.54^∗^	1.76^∗^	1.52^∗^	1.68^∗^	1.45^∗^	1.37^∗^	1.51^∗^	1.82^∗^	2.09^∗^
C20:3n3	0.39	0.73	0.92	1.87^∗^	1.69^∗^	1.51^∗^	0.82	1.67	0.46	1.45	1.28	0.83	0.76	0.71	1.09	1.00	0.85	0.64

Note: M1 = month 1; M2 = month 2; M3 = month 3; Asterisks = significant (*p* < 0.05) differences in individual milk FA among the different treatments along the same row; green fonts = increasing trend throughout 3 months; red fonts = decreasing trend throughout 3 months; blue fonts = increasing trend; black fonts = decreasing trend; C14:1 = myristoleate; C16:1 = palmitoleate; C17:1 = *cis*-10-heptadecenoate; C18:1n9t = elaidiate; C18:1n9c = oleate; C20:1 = *cis*-11-eicosenoate; C18:2n6t = linolelaidate; C18:2n6c = linoleate; C20:3n3 = *cis*-11, 14, 17-eicosatrienoic.

**Table 6:** Classification and colour-coding of total FA contents (mean percentage of total FA) in milk from goats fed with different DPP supplementation at their respective months.

		Treatments																	
		Control			Ajwa DPP						Mariami DPP								
Fatty acid classification		—			10 g			20 g			10 g			20 g			30 g		
		M1	M2	M3	M1	M2	M3	M1	M2	M3	M1	M2	M3	M1	M2	M3	M1	M2	M3
Length	SCFA	14.41	16.33	16.69	13.74	16.30	14.32	17.74^∗^	17.69^∗^	15.93^∗^	12.58	14.30	13.50	13.58	14.95	17.22	13.74	14.35	13.37
	MCFA	51.03^∗^	51.65^∗^	48.83^∗^	47.23	50.52	46.10	49.60^∗^	47.70^∗^	48.88^∗^	48.24	45.81	50.61	45.69	43.15	48.01	42.32	44.75	44.20
	LCFA	34.55	32.01	34.48	39.03	33.22	40.58	32.66	34.61	35.26	39.11	40.06	35.89	40.73	41.90	34.76	43.94^∗^	40.90^∗^	42.43^∗^

Saturation	SFA	72.96^∗^	75.38^∗^	74.69^∗^	69.67	73.82	68.94	74.50	70.13	71.35	68.32	69.72	71.24	69.08	70.01	74.22	66.43	67.91	67.06
	MUFA	24.96	22.50	23.10	27.00	23.20	29.72	23.32	26.67	27.18	28.58	27.41	26.25	28.35	27.67	23.22	30.89^∗^	29.27^∗^	30.07^∗^
	PUFA	2.08	2.12	2.21	3.33	3.01	2.34	2.18	3.20	1.54	3.03	3.04	2.50	2.57	2.31	2.56	2.68	2.82	2.87

*Σ* Omega-6		1.61	1.35	1.24	1.25	1.27	1.00	1.36	1.46	1.10	1.58	1.76	1.64	1.85	1.65	1.47	1.69^∗^	1.98^∗^	2.22^∗^
*Σ* Omega-3		0.39	0.73	0.92	1.87^∗^	1.69^∗^	1.51^∗^	0.82	1.67	0.46	1.45	1.28	0.83	0.76	0.71	1.09	1.00	0.85	0.64
n-6/n-3 ratio		3.41^∗^	1.85^∗^	1.38^∗^	0.71	0.76	0.76	1.66	0.83	2.21	1.09	1.37	1.90	2.30	2.13	1.35	1.70	2.33	3.45

Note: M1 = month 1; M2 = month 2; M3 = month 3; Asterisks = significant (*p* < 0.05) differences in various classifications of total milk FA among the different treatments along the same row; green fonts = increasing trend throughout 3 months; red fonts = decreasing trend throughout 3 months; blue fonts = increasing trend; black fonts = decreasing trend; SCFA = short-chain FA; MCFA = medium-chain FA; LCFA = long-chain FA; SFA = saturated FA; MUFA = monounsaturated FA; PUFA = polyunsaturated FA; *Σ* = total.

**Table 7:** Summary of Ajwa and Mariami PLS model characteristics.

Model	PLS model characteristics	Value	RMSE
A vs. C	*R* ^2^	0.848	3.212
	*Q* ^2^	0.267	7.074
M vs. C	*R* ^2^	0.829	4.530
	*Q* ^2^	0.525	7.871

Note: A = Ajwa DPP; M = Mariami DPP; C = control; *R*^2^ = calibration; *Q*^2^ = cross-validation; RMSE = root mean squared error.

## Data Availability

The data that support the findings of this study are available from the corresponding author upon reasonable request.

## References

[1] Sharifi M., Bashtani M., Naserian A. A., Farhangfar H. (2015). The effect of feeding low quality date palm (*Phoenix dactylifera L.*) on the performance, antioxidant status and ruminal fermentation of mid-lactating Saanen dairy goats. *Small Ruminant Research*.

[2] Nudda A., Battacone G., Neto O. B. (2014). Feeding strategies to design the fatty acid profile of sheep milk and cheese. *RevistaBrasileira de Zootecnia*.

[3] Russo G. L. (2009). Dietary *n* − 6 and *n* − 3 polyunsaturated fatty acids: from biochemistry to clinical implications in cardiovascular prevention. *Biochemical Pharmacology*.

[4] Ward R. J., Travers M. T., Richards S. E. (1998). Stearoyl-CoA desaturase mRNA is transcribed from a single gene in the ovine genome. *Biochimica et Biophysica Acta (BBA)-Lipids and Lipid Metabolism*.

[5] Haug A., Hostmark A. T., Harstad O. M. (2007). Bovine milk in human nutrition: a review. *Lipids Health Disease*.

[6] Williams C. M. (2000). Dietary fatty acids and human health. *INRA Annual Zootechnology*.

[7] Chilliard Y., Ferlay A., Mansbridge R. M., Doreau M. (2000). Ruminant milk fat plasticity: nutritional control of saturated, polyunsaturated,transand conjugated fatty acids. *INRA Annual Zootechnology*.

[8] Salvamani S., Baskaran G., Shukor M. Y., Abu Bakar M. Z., Ahmad S. A. (2016). Phytochemical investigation, hypocholesterolemic and anti-atherosclerotic effects of Amaranthus viridis leaf extract in hypercholesterolemia-induced rabbits. *RSC Advances*.

[9] Hu F. B., Manson J. E., Willett W. C. (2001). Types of dietary fat and risk of coronary heart disease: a critical review. *Journal of American College of Nutrition*.

[10] Sabikhi L. (2004). Designer milk: an imminent milestone in dairy biotechnology. *Current Science*.

[11] Afiq M. A., Rahman R. A., Man Y. C., Al-Kahtani H. A., Mansor T. S. (2013). Date seed and date seed oil. *International Food Research Journal*.

[12] Hossain M. Z., Waly M. I., Singh V., Sequeira V., Rahman M. S. (2014). Chemical compositions of date-pits and its potential for developing value-added product - a review. *Polish Journal of Food and Nutrition Sciences*.

[13] Al-Shahib W., Marshall R. J. (2003). Fatty acid content of the seeds from 14 varieties of date palm Phoenix dactylifera L. *International Journal of Food Science and Technology*.

[14] Nehdi I., Omri S., Khalil M. I., Al-Resayes S. I. (2010). Characteristics and chemical composition of date palm (*Phoenix canariensis*) seeds and seed oil. *Industry Crops Products*.

[15] El Hadramiand A., Al-Khayri J. M. (2012). Socioeconomic and traditional importance of date palm. *Emirates Journal of Food Agriculture*.

[16] Martinez Marin A. L., Gomez-Cortes P., Castro A. G. G. (2012). Short communication: linear discriminant analysis and type of oil added to dairy goat diets. *Journal of Dairy Science*.

[17] Bassbasi M., Platikanov S., Tauler R., Oussama A. (2014). FTIR-ATR determination of solid non fat (SNF) in raw milk using PLS and SVM chemometric methods. *Food Chemistry*.

[18] Dervilly-pinel G., Courant F., Chéreau S. (2012). Metabolomics in food analysis: application to the control of forbidden substances. *Drug Test Analysis*.

[19] Min B. R., Hart S. P., Sahlu T., Satter L. D. (2005). The effect of diets on milk production and composition, and on lactation curves in pastured dairy goats. *Journal of Dairy Science*.

[20] Abd Rahman M. R., Hassan Z., Hassan M. S., Hashim R., Wong L. S., Syd Jaafar S. H. (2022). Multi-nutrient milk quality analysis applying chemometrics: a supplementation-based approach using dairy goats. *Journal of Advanced Research in Applied Sciences and Engineering Technology*.

[21] SydJaafar S. H., Hashim R., Hassan Z., Arifin N. (2018). A comparative study on physicochemical characteristics of raw goat milk collected from different farms in Malaysia. *Tropical and Life Sciences Research*.

[22] Mestawet T. A., Girma A., Ådnøy T., Devold T. G., Narvhus J. A., Vegarud G. E. (2012). Milk production, composition and variation at different lactation stages of four goat breeds in Ethiopia. *Small Ruminant Research*.

[23] Indarti E., Abdul Majid M. I., Hashim R., Chong A. (2005). Direct FAME synthesis for rapid total lipid analysis from fish oil and cod liver oil. *Journal of Food Composition and Analysis*.

[24] Sawaya W. N., Khalil J. K., Safi W. J. (1984). Chemical composition and nutritional quality of date seeds. *Journal of Food Sciences*.

[25] Devshony S., Etesholaand E., Shanib A. (1992). Characteristics and some potential applications of date palm (Phoenix dactyliferaL.) seeds and seed oil. *Journal of the American Oil Chemists' Society*.

[26] Gilmore L. A., Walzem R. L., Crouse S. F. (2011). Consumption of high-oleic acid ground beef increases HDL-cholesterol concentration but both high- and low-oleic acid ground beef decrease HDL particle diameter in normocholesterolemic men^1,2^. *Journal of Nutrition*.

[27] Besbes S., Blecker C., Deroanne C., Lognay G., Drira N. E., Attia H. (2005). Heating effects on some quality characteristics of date seed oil. *Food Chemistry*.

[28] Barreveld W. H. (1993). Date palm products. *FAO Agricultural Services Bulletin, No. 101, Rome, Italy*.

[29] Al-Shahib W., Marshall R. J. (2003). The fruit of the date palm: its possible use as the best food for the future. *International Journal of Food Science and Nutrition*.

[30] Arnould V. M. R., Soyeurt H. (2009). Genetic variability of milk fatty acids. *Journal of Applied Genetics*.

[31] Andreotti G., Lamanna R., Trivelloneand E., Motta A. (2002). 13C-NMR spectra of TAG: an easy way to distinguish milks from different animal species. *Journal of American Oil and Chemistry Society*.

[32] Martinez Marin A. L., Gomez-Cortes P., Castro A. G. G. (2012). Effects of feeding increasing dietary levels of high oleic or regular sunflower or linseed oil on fatty acid profile of goat milk. *Journal of Dairy Science*.

[33] Chilliard Y., Glasser F., Ferlay A., Bernard L., Roueland J., Doreau M. (2007). Diet, rumen biohydrogenation and nutritional quality of cow and goat milk fat. *Journal of Lipid Science Technology*.

[34] Fievez V., Vlaeminck B., Jenkins T., Enjalbertand F., Doreau M. (2007). Assessing rumen biohydrogenation and its manipulation in vivo, in vitro and in situ. *European Journal of Lipid Science Technology*.

[35] Noakes M., Nestel P. J., Clifton P. M. (1996). Modifying the fatty acid profile of dairy products through feedlot technology lowers plasma cholesterol of humans consuming the products. *American Journal of Clinical Nutrition*.

[36] Morsy T. A., Kholif S. M., Kholif A. E., Matloup O. H., Salem A. Z. M., Elella A. A. (2015). Influence of sunflower whole seeds or oil on ruminal fermentation, milk production, composition and fatty acid profile in lactating goats. *Asian-Australasian Journal of Animal Sciences*.

[37] Osmari E. K., Cecato U., Macedo F. A. F., Souza N. E. (2011). Nutritional quality indices of milk fat from goats on diets supplemented with different roughages. *Small Ruminant Research*.

[38] Adeyemi K. D., Sazili A. Q., Ebrahimi M. (2015). Effects of blend of canola oil and palm oil on nutrient intake and digestibility, growth performance, rumen fermentation and fatty acids in goats. *Animal Science Journal*.

[39] Ferlay A., Martin B., Pradel P. H., Coulon J. B., Chilliard Y. (2006). Influence of grass-based diets on milk fatty acid composition and milk lipolytic system in tarentaise and montbeliarde cow breeds. *Journal of Dairy Science*.

[40] de Vries M. J., Veerkamp R. F. (2000). Energy balance of dairy cattle in relation to milk production variables and fertility. *Journal of Dairy Science*.

[41] Al-Suwaiegh S. B. (2016). Effect of feeding date pits on milk production, composition and blood parameters of lactating Ardi goats. *Journal of Animal Science*.

[42] Bernard L., Rouel J., Leroux C. (2005). Mammary lipid metabolism and milk fatty acid secretion in alpine goats fed vegetable lipids. *Journal of Dairy Science*.

[43] Chilliardand Y., Ferlay A. (2004). Dietary lipids and forages interactions on cow and goat milk fatty acid composition and sensory properties. *Reproduction and Nutritional Development*.

[44] Davis S. R., Farr V. C., Prosser C. G. (2004). MilkL-lactate concentration is increased during mastitis. *Journal of Dairy Research*.

[45] Vlaeminck B., Fievez V., Cabrita A. R. J., Fonseca A. J. M., Dewhurst R. J. (2006). Factors affecting odd- and branched-chain fatty acids in milk: a review. *Animal Feed Science Technology*.

[46] Strzałkowska N., Jóźwik A., Bagnicka E. (2009). Chemical composition, physical traits and fatty acid profile of goat milk as related to the stage of lactation. *Animal Science Papers and Reports*.

[47] Kishino S., Takeuchi M., Si-Bum P. (2013). Polyunsaturated fatty acid saturation by gut lactic acid bacteria affecting host lipid composition. *Proceedings of the National Academy of Sciences*.

[48] Silanikove N., Leitner G., Merin U., Prosser C. G. (2010). Recent advances in exploiting goat's milk: quality, safety and production aspects. *Small Ruminant Research*.

[49] Zhang H., Ao C. J., Khas-Erdene S. L. W., Zhang X. F. (2015). Effects of different model diets on milk composition and expression of genes related to fatty acid synthesis in the mammary gland of lactating dairy goats. *Journal of Dairy Science*.

[50] Jenness R. (1980). Composition and characteristics of goat milk: review 1968−1979^1^. *Journal of Dairy Science*.

[51] Haenlein G. F. W. (2004). Goat milk in human nutrition. *Small RuminantResearch*.

[52] Hu F., Furihata K., Ito-Ishida M., Kaminogawa S., Tanokura M. (2004). Nondestructive observation of bovine milk by NMR spectroscopy: analysis of existing states of compounds and detection of new compounds. *Journal of Agricultural and Food Chemistry*.

[53] Mather I. H., Keenan T. W. (1998). Origin and secretion of milk lipids. *Journal of Mammary Gland Biology Neoplasia*.

[54] Rukkwamsuk T., Geelen M. J. H., Kruip T. A. M., Wensing T. (2000). Interrelation of fatty acid composition in adipose tissue, serum, and liver of dairy cows during the development of fatty liver postpartum^1^. *Journal of Dairy Science*.

[55] Lu J., Fernandes E. A., Elizabeth A. (2013). Changes in milk proteome and metabolome associated with dry period length, energy balance and lactation stage in postparturient dairy cows. *Journal of Proteome Research*.

[56] Sordillo L. M., Contreras G. A., Aitken S. L. (2009). Metabolic factors affecting the inflammatory response of periparturient dairy cows. *Animal Health Research Reviews*.

[57] Wood L. G., Scott H. A., Garg M. L., Gibson P. G. (2009). Innate immune mechanisms linking non-esterified fatty acids and respiratory disease. *Progress in Lipid Research*.

[58] Das Purkayastha M., Kalita D., Das V. K., Mahanta C. L., Ashim J., Thakur M. K. C. (2012). Effects of L-ascorbic acid addition on micro-filtered coconut water: preliminary quality prediction study using ^1^H-NMR, FTIR and GC-MS. *Innovation and Food Science Emerging Technology*.

[59] Dewhurst R. J., Shingfield K. J., Lee M. R. F., Scollan N. D. (2006). Increasing the concentrations of beneficial polyunsaturated fatty acids in milk produced by dairy cows in high-forage systems. *Animal and Feed Science Technology*.

[60] Poti P., Pajor F., Bodnár A., Penkszaand K., Köles P. (2015). Effect of micro-alga supplementation on goat and cow milk fatty acid composition. *Journal of Agricultural Research*.

[61] Kholif S. M., El-Shewy A. A., Morsy T. A., Abd El-Rahman H. H. (2015). Variations in protein and fat contents and their fractions in milk from two species fed different forages. *Journal of animal physiology and animal nutrition*.

[62] Simopoulos A. P. (2002). The importance of the ratio of omega-6/omega-3 essential fatty acids. *Biomedical Pharmacotherapy*.

[63] Simopoulos A. P. (2006). Evolutionary aspects of diet, the omega-6/omega-3 ratio and genetic variation: nutritional implications for chronic diseases. *Biomedical Pharmacotherapy*.

[64] Kim H. K., Choi Y. H., Verpoorte R. (2010). NMR-based metabolomic analysis of plants. *Nature Proteomics*.

[65] Courant F., Antignac J. P., Dervilly-pinel G., Bizec B. L. (2014). Basics of mass spectrometry based metabolomics. *Proteomics*.

[66] Arifah M. F., Nisa K., Windarsih A., Rohman A. (2022). The application of FTIR spectroscopy and chemometrics for the authentication analysis of horse milk. *International Journal of Food Science*.

[67] Blasko J., Kubinec R., Pavlıkov E., Krupık J., Sojak L. (2008). On the chemometric deconvolution of gas chromatographically unseparated trans-7,cis-9, cis-9,trans-11 and trans-8,cis-10 octadecanoic acid isomers in ewe and cow milks. *Journal of Food and Nutrition Research*.

[68] Myrzakozha D., Turgaliev D., Sato H. (2014). Determination of fatty-acid composition in oils of animal origin by near-infrared spectroscopy. *Food and Nutrition Sciences*.

[69] Caboni P., Manis C., Ibba I., Contu M., Coroneo V., Scano P. (2017). Compositional profile of ovine milk with a high somatic cell count: a metabolomics approach. *International Dairy Journal*.

[70] Wold S., Sjostrom M., Eriksson L. (2001). PLS-regression: a basic tool of chemometrics. *Chemometrics and Intelligent Laboratory Systems*.

[71] Triba M. N., Le Moyec L., Amathieu R. (2015). PLS/OPLS models in metabolomics: the impact of permutation of dataset rows on the K-fold cross-validation quality parameters. *Molecular BioSystems*.

[72] Bai Y., Zhang B., Zhang X. (2022). Discrimination between organic and conventional raw and UHT milk by fatty acid profile in Inner Mongolia,China. *International Journal of Dairy Technology*.

